# Experimental and Computational Approaches to Measure Telomere Length: Recent Advances and Future Directions

**DOI:** 10.1007/s11899-023-00717-4

**Published:** 2023-11-10

**Authors:** Alejandro Ferrer, Zachary D. Stephens, Jean-Pierre A. Kocher

**Affiliations:** 1 Division of Hematology, Mayo Clinic, Rochester, 200 First Street SW, Rochester, MN USA; 2 Center for Individualized Medicine, Mayo Clinic, Rochester, MN USA; 3 Quantitative Health Sciences, Mayo Clinic, Rochester, MN USA

**Keywords:** Telomere, Short read sequencing, Long read sequencing, Telomere restriction fragment, Single telomere absolute-length rapid assay, Telomere combing assay, Optical genome mapping

## Abstract

**Purpose of Review:**

The length of telomeres, protective structures at the chromosome ends, is a well-established biomarker for pathological conditions including multisystemic syndromes called telomere biology disorders. Approaches to measure telomere length (TL) differ on whether they estimate average, distribution, or chromosome-specific TL, and each presents their own advantages and limitations.

**Recent Findings:**

The development of long-read sequencing and publication of the telomere-to-telomere human genome reference has allowed for scalable and high-resolution TL estimation in pre-existing sequencing datasets but is still impractical as a dedicated TL test. As sequencing costs continue to fall and strategies for selectively enriching telomere regions prior to sequencing improve, these approaches may become a promising alternative to classic methods.

**Summary:**

Measurement methods rely on probe hybridization, qPCR or more recently, computational methods using sequencing data. Refinements of existing techniques and new approaches have been recently developed but a test that is accurate, simple, and scalable is still lacking.

## Introduction

Telomeres are non-coding hexamer repeats located at the ends of eukaryotic chromosomes. They protect the genome from enzymatic degradation, and the loss of distal DNA that naturally occurs due to the end-replication problem, which would eventually lead to information loss due to the inability of the replication process to copy entire linear DNA molecules [1–3]. Average telomere length (TL) in humans ranges from 8 to 15 kb at birth and is reduced by 50 to 200 bp in healthy normal somatic cells after each cell division [1–3]. Once TL reaches a critical threshold (the Hayflick limit), cells become senescent and lose their capacity to replicate [3, 4]. TL has long been recognized as a biomarker for chronological cellular aging and over the ensuing years has also become a biomarker for a broad variety of diseases and conditions (e.g., cancer, infertility, diabetes, and cardiovascular disease) [1, 5–8]. Pathogenic variants in telomere maintenance genes may likewise result in abnormally shortened telomeres and cause multisystemic diseases collectively termed telomere biology disorders (TBD) that include idiopathic pulmonary and liver fibrosis, bone marrow failure and cancer predisposition among other manifestations [9–13].

Many approaches have been developed to measure TL, but no single method is at the same time accurate, simple and scalable [14, 15]. The terminal restriction fragment (TRF) assay is a southern blot-based method for measuring TL with high accuracy, but it is labor-intensive, technically difficult, and thus not suitable for large studies [16–18]. Fluorescence in situ hybridization (FISH) and its variation adapted to flow cytometry (flowFISH) is one of the most successful alternatives to TRF. FlowFISH measures average TL in blood samples and, in combination with different cell surface markers, can detect TL in different cell populations [19]. This method is scalable and easier to use than TRF, and it is the only test approved for clinical use according to the clinical laboratory improvement amendments (CLIA). Nevertheless, its broad implementation is limited by the need to identify enough control samples to define normal ranges, and therefore, it is only available in a small number of institutions (Johns Hopkins Laboratories and RepeatDx) [19]. Additionally, flowFISH is challenging to perform in non-hematological tissues, which can be an issue in some studies as TL in blood may not be representative of TL in other organs. Another approach uses qPCR to measure average TL [20, 21]. The latter are amongst the most commonly used, as they are simple to implement, scalable, and compatible with any tissue source [20, 21]. However, one limitation with these approaches is that they have proven to be irreproducible for TL accuracy from laboratory to laboratory due to the inherent variability of qPCR [20, 21]. Finally, with the increasing incorporation of sequencing data into research and clinical practice, several computational methods have been developed to estimate TL from this data [22]. Approaches using short read sequencing are scalable and compatible with DNA from any tissue source but are generally limited to reporting average TL only. Approaches using long read sequencing are capable of reporting the lengths of individual telomeres but are costly and still in their early developmental stages, requiring further research and validation to be translated into clinical practice.

**Fig. 1:**
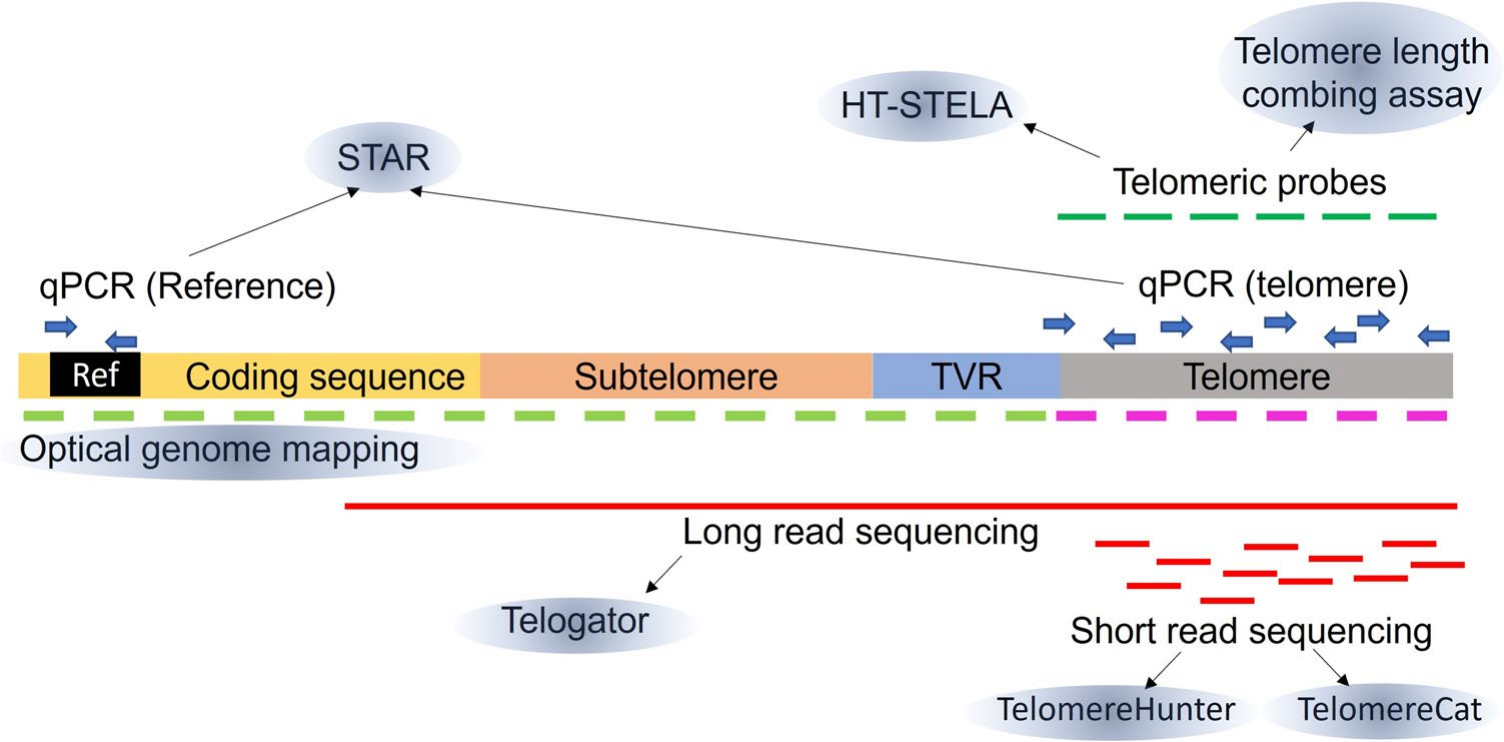
Schematic representation of the different telomere measurement techniques described in the text, indicating targeted region of the telomere in each case. STAR: single telomere absolute-length assay; HT-STELA: high-throughput single telomere length assay; Ref: reference; TVR: telomere variant repeat

In recent years, several refinements to these methods have been described; thus, it is critical to understand the advantages and limitations of these techniques in order to select the most appropriate for a given use case. In this review, we summarize the latest advances to measure TL using both bench-based and computational-based procedures (Fig. 1). We highlight the strengths and weaknesses of each technique (Table 1), provide guidance to select the most appropriate method, and describe how to interpret the different results.

**Table 1:** Tabulated summary of advantages and disadvantages of recent methods to measure telomere length

Method	Advantages	Disadvantages	Average TL	TL distribution	Chromosome arm-specific TL	References
HT-STELA	• Higher throughput than classic STELA • Used successfully to study several conditions	• More labor intensive than other options • Available for a limited number of chromosome arms			X*	[25, 26]
TCA	• High range of measurement (< 1–80 kb) • Can be automated • Measures TL distribution and average	• Requires specialized equipment • Time-consuming and costly • Lack of standardization has resulted in TL discrepancies between laboratories	X	X		[27]
Optical mapping	• Characterizes large DNA molecules to infer TL and subtelomere structure of individual telomeres • High throughput	• Requires specialized equipment • May miss telomeres from certain chromosome arms	X	X	X	[28]
STAR	• High range of measurement (02–320 kb) • High throughput • Measures TL distribution and average • Fast (~ 3 h) • Requires low DNA quantity	• Recently developed method still not broadly accepted	X	X		[35]
DNAmTL	• Robust and high throughput	• Not measuring TL directly but estimated from correlations • Only developed for lymphocyte TL	X**			[44, 45]
Short read sequencing	• High throughput • Can be used on existing datasets	• Limited to reporting average TL • Results may vary with NGS method employed	X			[22, 46–48]
Long read sequencing	• High throughput • Measures TL of most chromosome arms • Provides full telomere and subtelomeric sequence for further study	• High cost • Requires large DNA quantity • Measurable TL is limited by read length • Still in early developmental stages	X	X	X	[51]

*Only developed for a limited number of chromosome arms. **TL is estimated from a previously stablished correlation

*TL* telomere length, *HT-STELA* high-throughput single telomere length analysis, *TCA* telomere length combing assay, *STAR* single telomere absolute-length rapid assay, *NGS* next-generation sequencing

## Telomere Structure and Considerations of Telomere Measurement

Healthy human cells contain 92 telomeres (23 chromosome pairs, 2 arms per chromosome) each with its own length [1–3]. This telomeric DNA is comprised of TTAGGG hexamer repeats, also referred to as “canonical” repeats. Adjacent to telomeres are subtelomeres, informally defined as the most distal 500 kb of each chromosome arm. Subtelomeres are highly variable regions with a high frequency of structural rearrangements observed across samples [1–3]. In between the telomeres and subtelomeres are telomere variant repeat (TVR) regions, which are typically 1–2 kb in length and contain variations of TTAGGG repeats, often modified by a single base substitution. The variable length of TVR regions introduces uncertainty in many methods for measuring TL and is often accounted for in the form of a corrective factor. Applying different TL measurement methods to the same sample may result in different TL estimations, as methods such as qPCR or FISH that estimate the length of the canonical sequence differ from approaches such as TRF that include the subtelomeric and TVR regions in their measurements, potentially overestimating TL. Finally, different measurement methods were designed to measure different features of telomeres. While most methods measure average length of all 92 telomeres (e.g., TRF and qPCR) [16–18, 20, 21], some techniques report the length distribution of all 92 telomeres (e.g., FISH), the number of shortened telomeres (e.g., TeSLA) [23], or telomere length of certain specific chromosome arms (e.g., STELA) [24].

## Hybridization-Based Methodologies

TRF was the first method developed to measure TL, and its usage has been foundational to studies of telomere biology since the 1990s. Its methodological details are described elsewhere [16–18], but in summary, genomic DNA is digested using restriction enzymes that retain telomeres intact; these fragments are then detected using a telomeric probe by southern blot. Telomeres are shown as a smear where average TL is identified by the most intense area in the lane. The main limitations of TRF are its requirements for high amounts of DNA (1–10 µg), a time-consuming and labor-intensive protocol, and low scalability. Additionally, TRF sensitivity decreases dramatically for very short telomeres (< 2 kb), and it is not able to identify the shortest telomeres in the sample [16–18]. Despite these limitations, TRF is still considered the “gold standard” for TL, and it has been the basis for later methods like single telomere length analysis (STELA) [24] and telomere shortest length assay (TeSLA) [23]. This section will describe recent improvements to this approach and will summarize other approaches that also depend on labeled telomeric probes to measure TL.

### High-Throughput STELA

STELA was one of the first methods adapted from TRF [24]. It offered for the first time the ability to measure TL from specific chromosome arms, as opposed to other methods that were limited to reporting averages. STELA introduces a PCR step that uses telomere linkers and primers specific to a subtelomeric region of selected chromosome arm to amplify single telomeres that are then detected by southern blot. Thus, similar to TRF, STELA is labor intensive and not suitable to study a large number of samples. To overcome these limitations, Norris et al. adapted STELA as a high-throughput protocol (HT-STELA). [25] In this approach, specific chromosome arm telomeres (e.g., XpYp and 17p) are amplified through PCR, but samples are resolved using capillary gel electrophoresis instead of southern blot. Average telomere length is then determined using specialized software (e.g., PROSize and Agilent).

This approach has been used in studies including hundreds of samples across several use cases such as in TBD diagnosis or in predicting treatment response in patients with chronic lymphocytic leukemia [25, 26]. However, HT-STELA still has many of the limitations inherited from both TRF and STELA, including requiring large amounts of DNA and the fact that not all chromosome arms have unique sequences and thus cannot be analyzed with this approach, limiting its use to a small number of chromosome arms [25].

### Telomere Combing Assay

The telomere length combing assay (TCA) measures TL using a telomere-specific peptide nucleic acid (PNA) probe over stretched DNA fibers on a glass cover slip [27]. Results can be analyzed manually or using an automated quantification software (provided by the Genomic Vision platform) for higher throughput testing. TCA measures individual telomeres and has a wide dynamic range (< 1 to > 80 kb) making it suitable for samples with both very short or very long telomeres (e.g., TBD and cancer, respectively) [27]. In this approach, absolute TL is estimated by using a constant stretching factor (2 kb/µm), and since individual telomeres are being measured, it also provides the distribution of TL [27]. TCA’s main advantages are simplicity, automation, and accuracy to measure all telomeres in the sample. However, it is limited by the need for specialized equipment, as well as its high cost and time-consuming protocol as compared to other methods.

### High-Throughput Optical Genome Mapping

Optical genome mapping (OGM) was developed as an alternative to cytogenetics to detect copy number variation and structural anomalies in the genome [28]. This technique uses fluorescent labels that bind to specific sequence motifs, followed by uncoiling of the DNA molecules in nanochannels to analyze structural variations in a genome-wide manner. In more recent years, this technology has been applied to the analysis of telomeres and subtelomeres, where TL is measured via the intensity of TTAGGG-specific labels. The specific chromosome end from which a molecule originated is determined via its pattern of GCTCTTC motifs [29–31]. While this method has been able to provide an unprecedented characterization of subtelomere haplotypes, it requires specialized equipment and expertise in the use of these technologies. Additionally, the reported TL on benchmark samples exhibit high variance and telomeres of some chromosome arms can be missed if the sample preparation induces DNA breakage at fragile sites in subtelomere regions [32]. Additional validation of this technique is needed before it can be widely implemented in the field.

## qPCR-Based Method for Telomere Length Measurement

Quantitative PCR approaches remain among the most cost-effective and scalable methods to measure average TL and thus are the most frequently used for large epidemiological studies [20, 21]. This method measures the quantity of telomeric DNA normalized to a single-copy gene, usually expressed as a *T*/*S* (telomere/standard) ratio that can be translated to absolute length using a reference sample with known TL [20, 21]. qPCR methods are flexible and can be used on any tissue source. Conversely, they exhibit high sensitivity to preanalytical (e.g., DNA isolation method) and analytical (e.g., PCR conditions) variables which lead to inconsistent results between laboratories and even between experiments within the same laboratory. Additionally, qPCR results can be confounded in situations where the copy number of the reference genes can vary (e.g., from aneuploidy or genetic instability). In order to address these limitations, some attempts have been made to develop guidelines on how to perform and report TL assessment by qPCR, as well as methodological refinements developed over the last years. In this section, we will present an overview of these guidelines as well as other recently developed qPCR-based techniques.

### Standardized Guidelines for TL Reporting Using qPCR

There is growing interest in developing standardized guidelines to perform and report TL measurements through qPCR. One of the most significant standardizations was put forth by the Telomere Research Network (TRN), an initiative from the National Institute of Aging and National Institute of Environmental Health Sciences to establish best practices for the measurement of TL in population-based studies [33]. Other researchers have also published similar guidelines [21, 34]. All of them agree on three main areas for designing and reporting qPCR TL results: (A) report sample details including type, storage, DNA extraction method, and integrity; (B) qPCR assay employed; and (C) data analysis. Detailed criteria on each of these can be found on the TRN website (https://trn.tulane.edu/). Despite these efforts at standardization, these guidelines have not been widely adopted by the research community, and qPCR remains a highly sensitive approach potentially yielding variable results even when attempts are made to follow best practices.

### Single Telomere Absolute-Length Rapid Assay

The single telomere absolute-length rapid (STAR) assay is a qPCR-based method to measure the absolute telomere length of single telomere molecules [35]. In this assay, telomeric repeat sequences in individual telomere molecules are measured using qPCR in nanoliter compartments in the presence of a double-stranded DNA binding dye. This allows real-time monitoring of the reaction kinetics in each individual compartment. Only compartments with telomere molecules show exponential increase in PCR product that is correlated to the TL of the initial telomere molecule. Different amplification kinetics in each compartment represent the heterogeneous distribution of TL in the tested sample [35]. This approach tackles several limitations of classic qPCR protocols like copy number variability of the standard genes or in the PCR reaction performance [35]. However, it requires specialized equipment, and to date, very few laboratories have used this approach, which makes difficult to predict its utility in practice.

## Computational-Based Approaches to Estimate Telomere Length

As the costs of generating sequencing data continue to decline and its usage in research and clinical settings grows, there has been an increasing interest in using this type of data to estimate TL. “Short read” sequencing works by breaking the target DNA into short 50–300 bp fragments (“reads”) that are then amplified by PCR and aligned to a reference genome [36–38]. This approach is not suited to sequence highly repetitive sequences and therefore telomeres cannot be studied at nucleotide-resolution from short reads [36–38]. In addition to the length and sequence complexity of telomeres themselves, the variability of adjacent subtelomere regions that are not well represented in early versions of the human genome reference sequence (e.g., GRCh37 or GRCh38) also introduce uncertainty during read alignment. These issues have been improved by two recent developments: (A) The development of “long read” sequencing that generates reads from 10,000 to 100,000 bp, is improving the capacity to align reads to repetitive genomic regions, including telomeres [39–42] and (B) the release of the “telomere-to-telomere” (T2T) reference assembly [43]. A gapless human genome reference that resolved ~ 8% of the human genome still lacking from CRCh38. The T2T reference provides almost 200Mbp of novel sequence with high accuracy and included for the first time high-quality sequences for subtelomeres. In this section, we will describe the attempts to leverage short read and long read sequencing to estimate different aspects of TL, as well as a different approach using methylation sequencing data.

### Short-Read Next-Generation Sequencing

TelSeq was one of the first methods developed to estimate TL from short read sequencing data. It has been extensively used in the field and methodological details have been described elsewhere [22]. TelSeq estimates TL by counting the number of reads with canonical telomere repeats (TTAGGG) and in some cases telomere variant repeats as well. Later methods designed around this principle differ primarily in how they discriminate reads originating from telomeres from other non-telomere regions such as interstitial telomere repeats [22, 44–46]. TelomereHunter [44] is a more recent tool that estimates average TL from counts of TTAGGG repeats, as well as telomere variant repeats TCAGGG, TGAGGG, and TTGGGG. Repeat composition and reference alignment position are used to categorize telomere repeats as intrachromosomal, subtelomeric and intratelomeric. Average TL is then computed from the intratelomeric repeats. These strategies were found to improve correlations of average TL with other methods such as qPCR and TRF. Other short read methods achieve similar filtering by using different combinations of repeats in tandem when querying reads for telomere sequences [47, 48].

### Long-Read Next-Generation Sequencing

Long read sequencing, also referred to as third-generation sequencing, has been gaining attention in recent years due to significant improvements in throughput and affordability. The most frequently used long read sequencing technologies by Pacific Biosciences (PacBio) and Oxford Nanopore (ONT) produce high quality reads that are 10–20 kb in length [39–42], long enough to span the entirety of human telomeres in most use cases.

Among these methods, Telogator [49] is the first one to report chromosome-specific TL in human using long reads, building on earlier approaches that demonstrated the viability of clustering long reads at telomere boundaries [50, 51]. Telogator leverages the T2T reference genome and identifies reads that originate from telomere regions based on their pattern of canonical repeats. These reads are then “anchored” in the subtelomeres using a sequence alignment procedure. The major advantage of Telogator is its ability to estimate individual TLs for most of the 46 chromosome arms individually [49].

Approaches such as Telogator can estimate, from a single experiment, average TL, chromosome-arm specific TL, TL distribution, and potentially TL at the allele level [49]. It also opens the possibility of studying the biological significance of genomic variation in the subtelomeric regions. Long read sequencing is more costly than short read approaches, and it is impractical to generate long reads exclusively to measure TL. In this sense, selectively enriching telomere regions prior to sequencing [52] has shown promise in further reducing the cost of long read sequencing via sample multiplexing while also potentially capturing all telomere alleles. Though the read lengths generated by this approach are comparatively shorter and may not be viable for all studies. There are also considerations for read quality, as certain long read protocols can have systematic sequencing artifacts that affect telomeres [53]. Another approach recently developed for ONT (Telo-seq) [54] uses adapters that bind to the 3′ single stranded overhand at the end of the chromosome. The 5′ end of these telomere adapters is complementary to the sequencing adapter, allowing for the sequencing of the full telomere and the subtelomeric region. The lower cost of ONT makes this an attractive approach that is likely to become a preferred option in the early future.

All these technologies, however, are still in the early stages of development and the accuracy of determining from which chromosome end a read originated remains challenging even with long reads. The variability and homology between subtelomeres in different chromosomes can result in telomere alleles being misassigned to incorrect chromosome arms resulting in inaccurate measurements. The high rate of structural variation in subtelomeres may manifest as substantial differences between a sample’s sequence and the T2T reference. We expect that this limitation may be mitigated over time, either by further increases in the length of the reads, or as additional high quality subtelomere assemblies from different populations become publicly available, like the T2T-YAO [55] and T2T-Han [56] assembly or the pangenome references [57].

The advantage of methods that use sequencing data is that they are easy to run on existing datasets and are scalable for large studies. However, it is still impractical and costly to generate sequencing data with the sole purpose of measuring TL. Therefore, their application has largely been limited to datasets created for other purposes (e.g., genetic variant analysis). Despite these limitations, the high-resolution view enabled by long read approaches potentially facilitates broader comparative studies of individual telomere alleles and their relationship to senescence or telomere dysfunction. For example, applications to aging or aging-related disorders could provide insight into whether certain conditions are driven by the shortest telomeres, a subset of shortened telomeres, or reduced averaged TL.

### DNA Methylation Estimator of Telomere Length (DNAmTL)

Similar to TL, methylation of cytosines in cytosine-phosphate-guanine dinucleotides (CpG) has emerged as an additional biomarker for aging. CpG methylation frequencies have formed the basis for machine learning algorithms to quantify “epigenetic clocks” with the goal of estimating the physiological age of any given sample. Using this approach, Dr. Lu et al. developed DNAmTL, an estimator that uses one of these biological clocks to estimate average TL in leukocytes using the methylation status of 140 CpGs [58, 59]. This method is very robust and outperformed leukocyte TL measured by TRF in several aspects, including correlation with age (*r* ~  − 0.75 for DNAmTL versus *r* ~ 0.35 for LTL), predicting time-to-death (*p* = 2.5E − 20), time to coronary heart disease (*p* = 6.6E − 5), time to congestive heart failure (*p* = 3.5E − 6), and association with smoking history (*p* = 1.21E − 17).

This method offers the clear advantage of using a single technique to measure at the same time two markers of aging (TL and CpG methylation). Nevertheless, this approach does not measure TL directly but, instead, offers an estimation of average TL using the predictive model developed by the authors [58, 59]. Therefore, in situations not resembling the training dataset, these estimations may not be completely accurate. For the same reason, these estimations are only valid for lymphocyte TL, needing to develop new models for TL estimation in other cell types.

## Conclusions

Several options to measure TL have been developed and improved over recent years, but the search continues for a simple and scalable assay that achieves at the same time accurate results. Each technique has different strengths and limitations that need to be carefully considered to match the purpose of the research undertaken. New bioinformatic tools able to provide TL with a level of granularity not previously possible could provide an attractive alternative to the experimental techniques [60, 61] but are still costly and in need of further refinements. We anticipate that declining cost of long read sequencing and the use of telomere-enrichment approaches prior to sequencing will contribute to broader implementation of these techniques, shaping the future of telomere research.

## Data Availability

Not applicable.
